# High-Sensitivity Cesium Aerosol Sensing for Nuclear Severe-Accident Monitoring by Integrating Laser-Induced Plasma RGB Imaging with Convolutional Neural Network (CNN) Analysis

**DOI:** 10.3390/s26185767

**Published:** 2026-09-11

**Authors:** Sung-Uk Choi, Chang Uk Koo

**Affiliations:** 1 Laser Technologies Group, Lawrence Berkeley National Laboratory, Berkeley, CA 94720, USA; 2 Pohang Accelerator Laboratory, Pohang University of Science and Technology, Pohang 37673, Republic of Korea

**Keywords:** cesium aerosol sensing, laser-induced plasma, RGB imaging, convolutional neural network

## Abstract

Rapid identification of radioactive cesium (Cs) aerosols is important for nuclear severe-accident monitoring. However, conventional analytical methods often require extended measurement times or complex instrumentation. Here, we present a sensitive and compact sensing approach that integrates laser-induced plasma RGB imaging with a convolutional neural network (CNN). A detection configuration was established in which laser irradiation generated plasma from Cs-containing aerosols within a flowing gas stream, and the resulting emission was directly captured using a CMOS camera. Rather than resolving individual emission lines, the CNN learned subtle Cs-dependent variations in the spatial and RGB intensity distributions of the plasma images. To simplify training under limited-data conditions, the model was designed to address a binary classification task, distinguishing Cs-negative conditions (normal) from Cs-positive conditions (abnormal). Predictions from 100 consecutive laser shots were aggregated to provide a sensing decision within 5 s. Using a criterion requiring Cs-positive classification in at least 99% of independent measurements, the operational limit of detection (LOD) was determined to be 0.03 μg/m^3^, approximately one order of magnitude lower than values reported for the closest comparable laser-based cesium aerosol measurements. These results demonstrate that plasma RGB imaging combined with a CNN algorithm can provide a compact, highly sensitive, and real-time platform for cesium aerosol monitoring.

## 1. Introduction

Nuclear power plant accidents can release volatile fission products from damaged fuel into the reactor coolant system, containment atmosphere, and ventilation or off-gas pathways [1]. As hot fission-product vapors move into cooler regions, they can nucleate, condense, or attach to pre-existing particles, thereby forming radioactive aerosols [2]. Cesium is of particular concern because accident-relevant compounds such as CsOH and CsI can volatilize at elevated temperatures and subsequently form or associate with hygroscopic airborne particles during cooling and transport [3,4,5]. If released into the environment and deposited, long-lived ^137^Cs can contribute to persistent contamination [6]. Rapid and continuous detection of cesium-containing aerosols is therefore important for identifying abnormal release conditions and supporting source-term assessment, containment evaluation, and off-gas treatment monitoring.

Airborne cesium can be monitored using either radiological measurements or elemental analytical methods [7,8]. Gamma-ray spectrometry identifies gamma-emitting cesium isotopes and therefore remains essential for radiological confirmation. At low airborne activity levels, however, reliable detection depends on counting statistics, background radiation, detector shielding, measurement geometry, and acquisition time [7]. Elemental techniques such as mass spectrometry provide high sensitivity but require aerosol collection, sample transfer, ionization, mass analysis, and vacuum instrumentation [8]. Consequently, their practical requirements motivate the development of a simpler sensor capable of rapidly screening changes in the elemental composition of a flowing aerosol stream. Such a sensor would complement, rather than replace, isotope-specific radiological monitoring.

Laser-induced plasma techniques are attractive for direct aerosol analysis because they can interrogate suspended particles within a flowing gas without prior filter collection or extensive sample preparation [9,10]. When a focused nanosecond laser pulse intercepts an aerosol particle, rapid energy deposition causes heating, vaporization, atomization, and ionization, producing a short-lived luminous plasma. In laser-induced breakdown spectroscopy (LIBS), the plasma emission is separated by wavelength using a spectrometer. Because individual elements emit characteristic atomic and ionic lines, the resulting spectrum can be used for elemental identification and, with suitable calibration, quantitative analysis [9,10]. LIBS has consequently been investigated for online and in situ aerosol measurements. Diaz et al. combined conditional single-shot analysis with aerosol-sampling statistics and reported a Ca concentration limit of detection of 0.45 μg/m^3^ [11]. Andrews et al. further demonstrated the simultaneous monitoring of Cs- and Rb-containing aerosols in a molten-salt-reactor surrogate off-gas stream using wavelength-resolved emission and partial least-squares regression [12]. More recently, LeCroy et al. demonstrated LIBS analysis of Cs and other fission-product surrogates in aerosolized LiCl–KCl salt [13], while CNN-assisted LIBS has also been applied to Cs analysis in salt-lake brine [14]. These studies demonstrate the elemental selectivity of aerosol LIBS but also highlight the challenge of detecting trace aerosol concentrations from weak element-specific emission.

Laser-induced breakdown detection (LIBD) uses a simpler, event-based measurement principle. Instead of resolving the wavelengths emitted by the plasma, LIBD records whether a laser pulse produces a breakdown event. When the pulse energy is maintained below the breakdown threshold of the carrier gas, a particle entering the laser focal region can facilitate localized ionization and generate a detectable optical or acoustic response [15,16]. Because the complete breakdown event is detected rather than the intensity of an individual emission line, LIBD can provide high particle-detection sensitivity using a relatively simple optical configuration. For example, a recent liquid-phase study reported a detection limit of approximately 8 × 10^4^ particles/mL for nanoparticles [16]. However, LIBD can indicate that a particle-triggered breakdown event has occurred but cannot inherently determine which chemical species caused it. This limitation creates an important sensing gap: LIBD can detect the presence of an aerosol particle but cannot independently distinguish a normal background aerosol from an abnormal Cs-containing aerosol.

One possible way to obtain composition-dependent information without a spectrometer is to record each plasma event as a color image. A red–green–blue (RGB) camera cannot resolve individual atomic emission lines, but its three broad color channels integrate different portions of the visible spectrum. Because the plasma emission is collected without wavelength dispersion, RGB imaging can retain high optical throughput, which is advantageous for detecting weak aerosol-induced plasma events. The image also preserves the two-dimensional size, shape, and spatial intensity distribution of the plasma. These spatial characteristics can also vary with aerosol composition and provide additional information for chemical discrimination.

Composition-dependent variations in broadband plasma emission may therefore appear as subtle changes in the RGB distribution, even when the composite images appear visually similar. Convolutional neural networks (CNNs) are well suited to this type of data because they jointly analyze color and spatial patterns rather than relying on an integrated intensity or channel ratio [17,18]. In this framework, we developed a compact and sensitive sensing platform that integrates aerosol-triggered laser-induced plasma, direct RGB imaging with a CMOS camera, and CNN-based analysis for detecting Cs aerosols. Specifically, we established a laser–aerosol interaction system in which a focused nanosecond Nd:YAG laser was directed into a flowing aerosol stream to generate localized plasma events. The resulting two-dimensional plasma emission was directly captured as RGB images without wavelength-resolved spectroscopic detection. A compact CNN was used to extract subtle composition-dependent color and spatial features from individual plasma images and generate an event-level Cs-likeness score. The event-level scores were aggregated over 100 consecutive laser shots to provide a binary sensing decision within 5 s. The operational limit of detection (LOD) was determined to be 0.03 μg/m^3^, defined as the lowest Cs concentration classified as Cs-positive in at least 99% independent measurements. This integrated framework combines the high event-detection sensitivity with image-based chemical discrimination, enabling rapid and sensitive Cs aerosol monitoring using a simplified optical configuration.

## 2. Materials and Methods

### 2.1. Laser-Induced Plasma Imaging System

**Figure 1:**
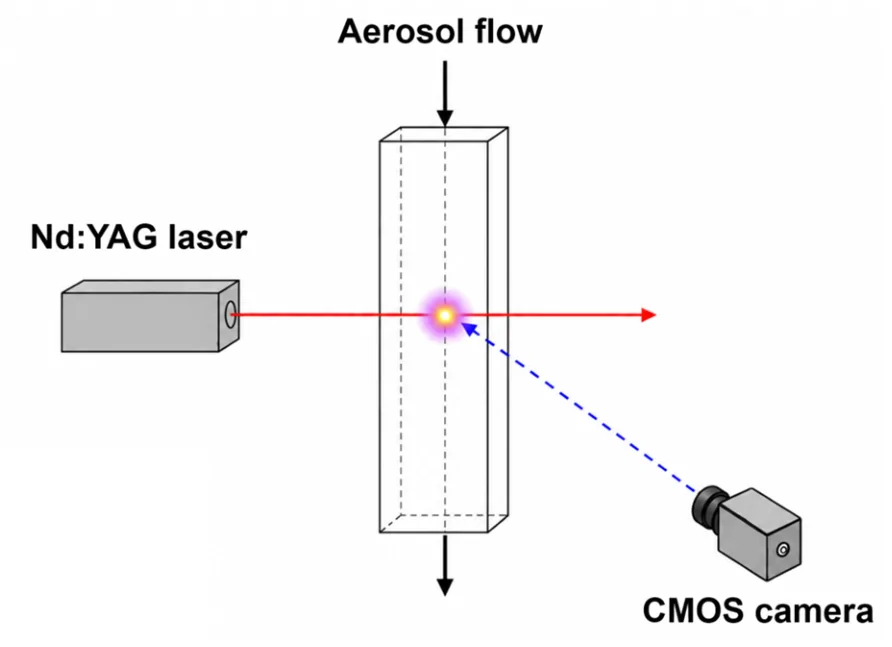
Schematic of the laser-induced plasma RGB imaging system for aerosol sensing. An aerosol stream was introduced into the interaction cell, where focused Nd:YAG laser pulses induced aerosol-triggered plasma formation. The resulting two-dimensional plasma emission was directly captured using a CMOS camera to obtain RGB images.

Figure 1 presents a schematic of the aerosol-generation and laser-induced plasma imaging system. A Q-switched Nd:YAG laser (Brilliant, Quantel, Brittany, France) delivered 1064 nm pulses with a pulse duration of less than 5 ns at a repetition rate of 20 Hz. The laser beam diameter at the entrance of the focusing lens was approximately 7 mm. The laser beam was focused into the center of an aerosol flow cell using a fused-silica lens with a focal length of 100 mm. The flow cell had an internal cross-section of 20 × 20 mm and a length of approximately 50 mm along the aerosol-flow direction. The laser pulse energy was adjusted using an external attenuator and measured at the entrance of the flow cell before and after each acquisition series.

Plasma emission was recorded at 90° relative to the laser axis using a global-shutter color CMOS camera (Basler ace, IMX287 color sensor, Ahrensburg, Germany) positioned 10.0 cm from the focal volume. According to the manufacturer-provided spectral response, the blue, green, and red channels are primarily sensitive over approximately 400–520 nm, 480–600 nm, and 560–680 nm, respectively.

The camera and laser Q-switch were synchronized so that one camera frame was acquired for each laser pulse. A delay of 1 μs was applied after the laser pulse to suppress the intense early-time continuum emission. The camera exposure time was fixed at 20 μs, which was selected to integrate most of the subsequent plasma emission because the plasma intensity decayed to the background level at approximately 18–19 μs.

The field of view was calibrated at the focal plane, and a 256 × 256-pixel region of interest centered on the plasma corresponded to an area of approximately 4 × 4 mm. Each RGB frame was dark-subtracted on a channel-by-channel basis. A frame was classified as plasma-positive when the integrated luminance within the predefined focal-volume region exceeded the mean dark-frame value by five standard deviations, and the thresholded image contained a connected component of at least nine pixels. These criteria were applied to reject detector noise and isolated hot pixels. The plasma position was determined from the intensity-weighted centroid of the largest valid connected component.

### 2.2. Aerosol Generation and Characterization

Nonradioactive cesium was used as a surrogate for radioactive Cs because it provides essentially identical chemical behavior and optical characteristics for the aerosol-generation and optical-detection processes considered in this study. CsOH was selected as the cesium-bearing precursor because cesium hydroxide is a representative soluble Cs compound relevant to severe nuclear reactor accidents [3]. LiOH was used to represent the normal coolant-chemistry background because it is routinely employed for pH control in pressurized-water reactors [19].

LiOH and CsOH stock solutions at a concentration of 0.10 mol/L were prepared using deionized water and reagent-grade LiOH and CsOH (Sigma-Aldrich, St. Louis, MO, USA). The solutions were aerosolized using two independently operated single-jet pneumatic atomizers (Model 9302, TSI, Shoreview, MN, USA). Each atomizer was operated with clean, dry N_2_ at an inlet pressure of approximately 25 psi (172 kPa), corresponding to a nominal aerosol output flow of approximately 6.5 L/min. The precursor solution was supplied from the atomizer reservoir by pneumatic aspiration rather than by an independently controlled liquid feed pump. The aerosol output from each generator was continuously produced, and a controlled fraction of each aerosol stream was introduced into the downstream dilution line, while the excess flow was exhausted.

The generated aerosol streams were passed separately through diffusion dryers filled with silica gel to remove residual water droplets. Additional dry N_2_ was subsequently introduced as dilution gas, and the conditioned LiOH and CsOH aerosol streams were independently adjusted using mass-flow controllers before being combined in a mixing chamber. The total gas flow entering the laser interaction cell was maintained at 3.0 L/min for all measurements. The relative contributions of the two aerosol streams were varied while maintaining the same total flow and total airborne alkali-metal concentration. The LiOH/CsOH composition series was established by adjusting the relative contributions of the two conditioned aerosol streams, while the resulting airborne concentrations were independently verified by ICP-MS.

The particle-size distribution downstream of the drying stage was characterized using a scanning mobility particle sizer (SMPS; TSI). The dried aerosol exhibited a count median diameter of approximately 220 nm with a geometric standard deviation of approximately 1.7 under the operating conditions used in this study. These measurements were consistent with the generation of predominantly submicrometer dried aerosol particles and indicated effective removal of large residual droplets.

The airborne Li and Cs mass concentrations delivered to the laser interaction cell were independently verified by filter collection followed by inductively coupled plasma mass spectrometry (ICP-MS). Aerosol samples were collected over a fixed sampling interval at the same flow condition used for the optical measurements, and the collected Li and Cs masses were converted to airborne concentrations using the measured sampling volume. The total airborne alkali-metal concentration was maintained at 0.50 μg/m^3^. This concentration was selected to represent the lower concentration range relevant to conventional aerosol laser-induced breakdown spectroscopy measurements [11,12]. The relative contributions of Li and Cs were varied from the LiOH-only condition to the CsOH-only condition, as summarized in Table 1. Throughout this study, the Cs concentration refers to the mass of elemental Cs per unit volume of sampled gas.

### 2.3. CNN Architecture and Training

The CNN was implemented in Python 3.11 using background-subtracted 256 × 256 × 3 RGB plasma images as input. The network consisted of three convolutional blocks with 32, 64, and 128 filters, respectively; each block included a 3 × 3 convolution, rectified linear unit activation, and 2 × 2 max pooling. Global average pooling was then applied, followed by a fully connected layer with 32 units and a single-output layer. The output was passed through a sigmoid function to generate an event-level Cs-likeness score.

The network was trained using binary cross-entropy with logits and the AdamW optimizer. The initial learning rate, weight decay, and batch size were 10^−3^, 10^−4^, and 32, respectively, with a maximum of 100 training epochs. Early stopping was applied when the validation loss failed to improve by at least 10^−4^ for ten consecutive epochs. Ten independently acquired runs from each end-member condition were divided at the run level into six training runs, two validation runs, and two test runs. The complete training procedure was repeated using 20 different run-level splits and random seeds to evaluate robustness to data partitioning and initialization.

No resizing or data augmentation was applied, and the original 256 × 256 RGB regions of interest were used directly for model training and inference. The mean inference time was 2.1 ms per image, and processing and aggregation of a 100-image block required approximately 0.22 s, substantially shorter than the 5 s required for acquisition of 100 laser shots at 20 Hz.

## 3. Results and Discussion

### 3.1. Optimization of Aerosol-Triggered Plasma Generation

**Figure 2:**
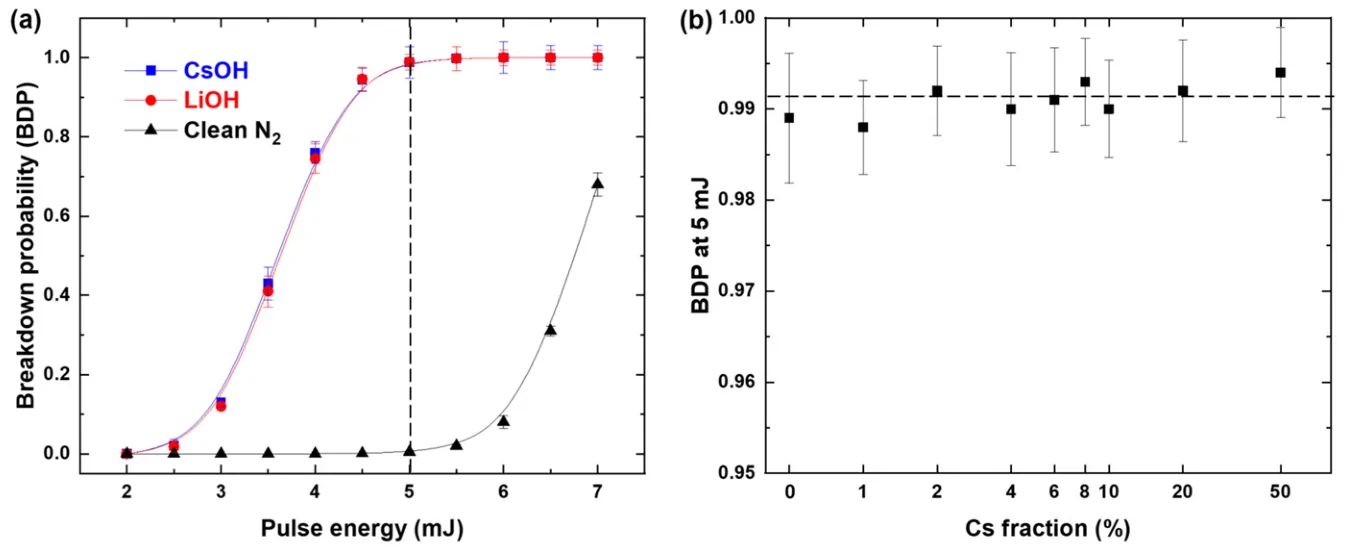
Aerosol-triggered plasma generation characteristics of LiOH and CsOH aerosols. (**a**) Breakdown probability (BDP) of aerosol-plasma generation as a function of laser pulse energy for LiOH aerosol, CsOH aerosol, and clean N_2_. The vertical dashed line indicates the selected operating energy of 5.0 mJ. (**b**) BDP of aerosol-plasma generation at 5.0 mJ as a function of Cs fraction at a constant total airborne alkali-metal concentration of 0.5 μg/m^3^. The horizontal dashed line indicates the mean BDP across the tested compositions. The nearly invariant BDP demonstrates that aerosol-plasma event counting alone provides little chemical selectivity between LiOH- and CsOH-containing aerosols.

Before analyzing composition-dependent information in the RGB plasma images, the laser pulse energy was optimized to generate frequent aerosol-triggered plasma events while suppressing breakdown of the surrounding carrier gas. This operating condition was essential because individual plasma events served as the input for the subsequent image analysis. Plasma occurrence was quantified using the breakdown probability (BDP), which is the principal analytical response in conventional laser-induced breakdown detection (LIBD). In LIBD, each laser shot is recorded as either a breakdown or a no-breakdown event. The BDP was calculated as the ratio of plasma-positive laser shots (N_plasma_) to the total number of laser shots (N_shot_): N_plasma_/N_shot_. Because one RGB frame was acquired synchronously with each laser pulse, each frame corresponded to a single laser shot. The laser pulse energy was varied from 2 to 7.0 mJ in increments of 0.5 mJ for clean N_2_, pure LiOH aerosol, and pure CsOH aerosol. As shown in Figure 2a, the energy scan clearly distinguished aerosol-triggered plasma formation from breakdown of the clean N_2_ carrier gas. Breakdown events became appreciable for both LiOH and CsOH aerosols above approximately 2 mJ, followed by a rapid increase in BDP with increasing pulse energy. At 5.0 mJ, the BDP reached 0.989 for LiOH and 0.994 for CsOH, whereas the BDP for clean N_2_ remained negligible. Increasing the pulse energy beyond 5.0 mJ produced little additional improvement in aerosol-event probability while increasing carrier-gas breakdown, plasma brightness, and the possibility of detector saturation. Accordingly, 5.0 mJ was selected as the operating energy for all subsequent RGB imaging measurements.

Notably, although the selected operating condition provided a high plasma-event yield, the BDP responses of the LiOH and CsOH aerosols were nearly indistinguishable. At 5.0 mJ, the absolute difference between the BDP values of the two pure aerosols was only approximately 0.01. Moreover, the BDP remained within the narrow range of 0.98–0.99 across the entire LiOH/CsOH composition range, as shown in Figure 2b. Thus, plasma-event counting could readily confirm the presence of aerosol particles but could not determine whether Cs was present in the LiOH background. This result highlights the intrinsic limitation of LIBD measurements. When plasma formation is represented only as a breakdown or no-breakdown event, the composition-dependent optical information contained in the plasma emission is discarded. The nearly invariant BDP response therefore demonstrated the need to analyze the emitted light itself. The composition-dependent information retained in the RGB plasma images is examined in Section 3.2.

### 3.2. Composition-Dependent RGB Signatures in Direct Plasma Images

**Figure 3:**
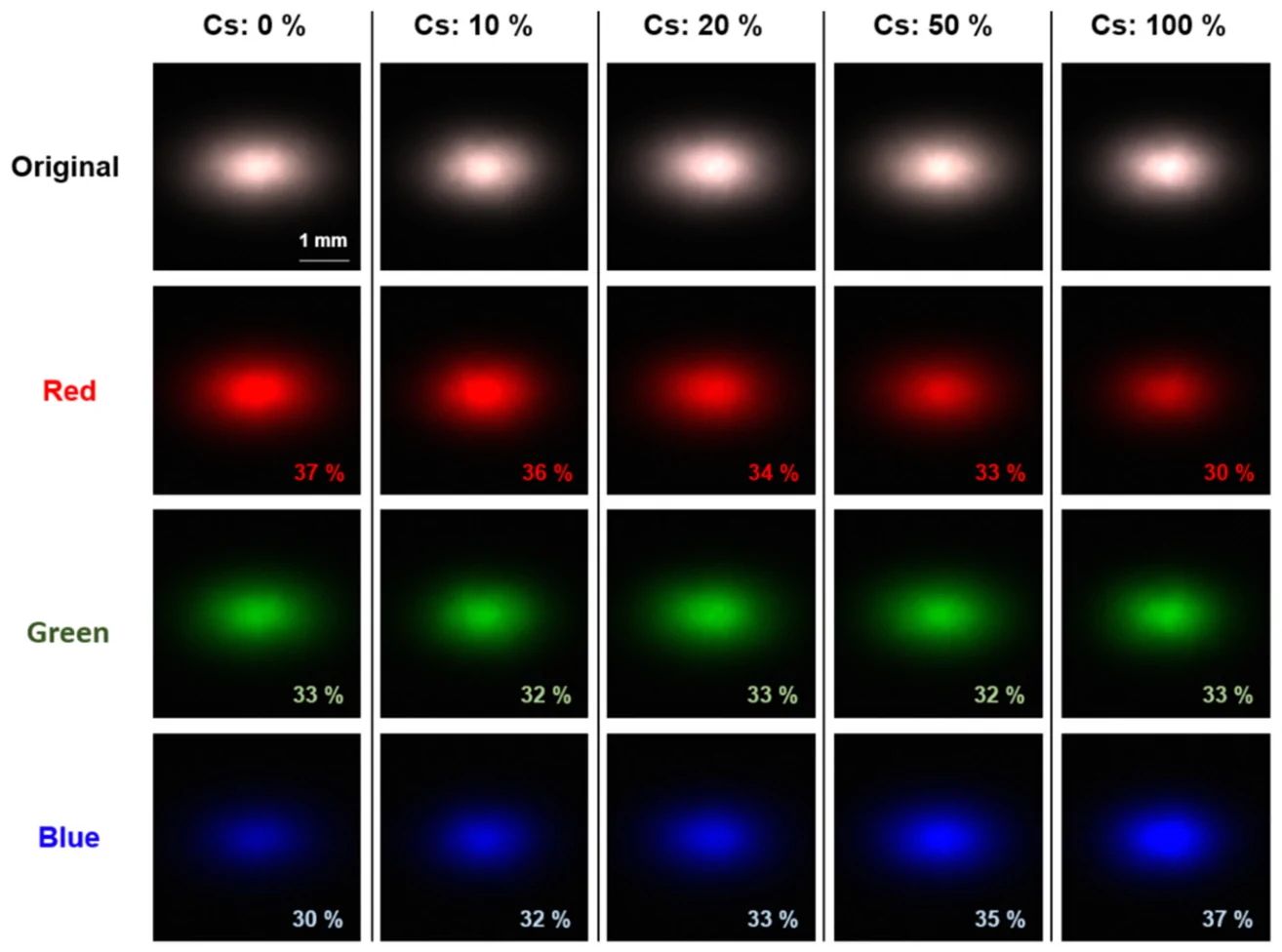
Composition-dependent RGB characteristics of plasma images. Representative images are shown for aerosol samples containing 0, 10, 20, 50, and 100% Cs. For each composition, the plasma-positive frame with an integrated RGB intensity closest to the median of the corresponding distribution was selected for visualization. The upper row shows the composite RGB images, and the lower rows show the separated red, green, and blue channels. The percentages indicate the spatially integrated intensity of each channel normalized to the summed RGB intensity. As the Cs fraction increased, the red-channel contribution decreased from 37% to 30%, whereas the blue-channel contribution increased from 30% to 37%; the green-channel contribution remained nearly constant at approximately 32–33%. The scale bar applies to all images.

The nearly invariant BDP response described in Section 3.1 demonstrated that plasma-event occurrence alone provided little chemical selectivity. We therefore examined whether the directly recorded plasma images retained composition-dependent information in their RGB distributions. Figure 3 presents representative plasma images for the aerosol samples corresponding to the Cs fractions listed in Table 1. For each composition, the frame with an integrated RGB intensity closest to the median of the corresponding plasma distribution was selected as the representative image. The composite images exhibited broadly similar plasma morphologies and overall color appearances across the entire composition range. This limited visual distinction can be attributed to the common aerosol and plasma background, together with the broadband continuum emission. Consequently, the presence of Cs could not be identified reliably through visual inspection of the composite plasma images alone.

To extract the color information retained by the camera, each plasma image was separated into its red, green, and blue channels in Figure 3. The intensity of each channel was spatially integrated over the plasma region and normalized to the summed RGB intensity. The percentages shown in Figure 3 represent these normalized channel contributions. A systematic redistribution of the RGB contributions was observed with increasing Cs fraction. For the 0% Cs sample, the normalized red, green, and blue contributions were 37%, 33%, and 30%, respectively. As the Cs fraction increased, the red contribution progressively decreased from 37% to 30%. In contrast, the blue contribution increased from 30% to 37%, while the green contribution remained nearly constant at approximately 32–33% throughout the composition series. Thus, increasing the Cs content produced opposing trends in the red and blue channels.

These trends are consistent with the characteristic atomic emissions of the two alkali elements. Li emissions near 610.4 and 670.8 nm can contribute to the red-sensitive region, whereas Cs emissions near 455.5–459.3 nm fall primarily within the blue-sensitive region. Although Cs has a strong resonance emission at 852.1 nm, this wavelength is strongly attenuated by the built-in IR-cut filter of the camera and is therefore expected to make only a minor contribution to the recorded RGB signal.

The observed increase in the blue-channel contribution cannot be uniquely attributed to the Cs emissions near 455–459 nm, because changes in broadband continuum emission and plasma conditions may also contribute to the RGB redistribution. In addition, because the total alkali-metal concentration was fixed, increasing Cs concentration was accompanied by a corresponding decrease in Li concentration. Therefore, the observed RGB redistribution and CNN response reflect the combined compositional change associated with increasing Cs and decreasing Li rather than an isolated Cs response.

Each RGB channel, however, covers a broad wavelength range and integrates broadband continuum emission together with multiple atomic and molecular features. The normalized channel contributions should therefore not be interpreted as line-resolved spectral intensities. Rather, they demonstrate that direct color imaging preserves a measurable composition-dependent redistribution of broadband plasma emission that cannot be identified from plasma occurrence or visual inspection alone. Because these differences were subtle and spatially distributed across the plasma images, a machine learning algorithm was subsequently employed to extract the combined color and spatial signatures of individual plasma events.

### 3.3. CNN-Based Plasma Image Analysis and Measurement-Level Classification

In nuclear severe-accident monitoring, the primary sensing objective is the rapid identification of a transition from a normal Cs-negative condition to an abnormal Cs-positive condition. Such monitoring must provide timely decisions despite the limited availability of labeled accident-relevant data and the variations in measurement conditions. Accordingly, the image-analysis task was formulated as a binary classification problem that distinguishes Cs-negative and Cs-positive plasma events. In this framework, a compact convolutional neural network (CNN) was employed to jointly extract color and spatial features from individual RGB plasma images, and the event-level predictions were subsequently aggregated over multiple laser shots to provide a robust measurement-level decision.

**Figure 4:**
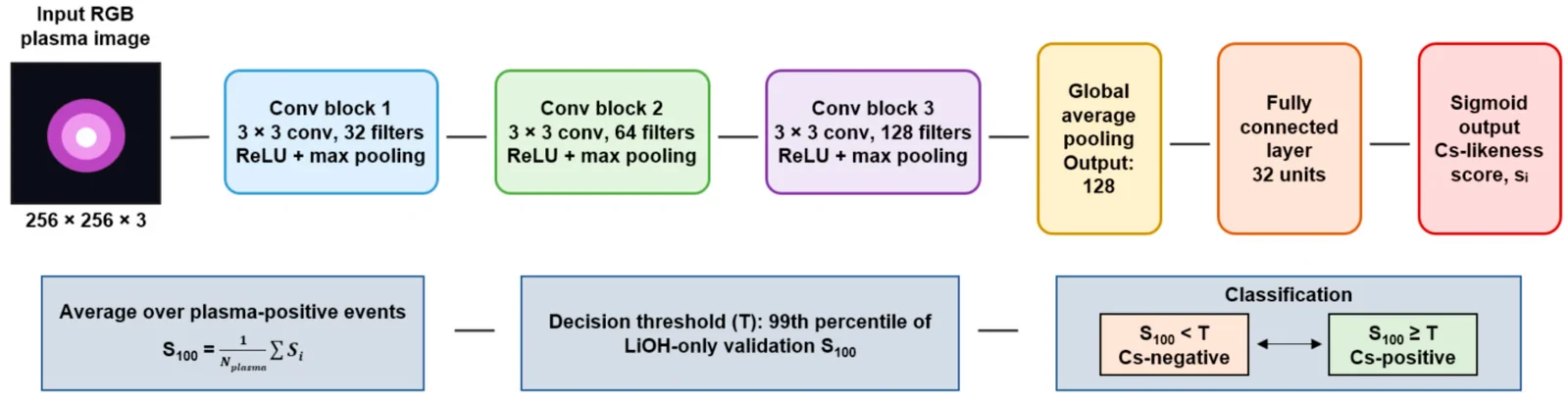
CNN architecture and measurement-level decision workflow for Cs aerosol detection. Each background-subtracted 256 × 256 × 3 RGB plasma image was processed through three convolutional blocks containing 32, 64, and 128 filters, respectively. Global average pooling converted the final feature maps into a 128-element vector, which was passed through a 32-unit fully connected layer and a sigmoid output to generate an event-level Cs-likeness score, *s_i_*. The scores of plasma-positive events within 100 consecutive laser shots were averaged to obtain the block-level score, S_100_. A decision threshold, T, derived from the LiOH-only validation distribution was then used to classify each measurement block as Cs-negative or Cs-positive.

The CNN was implemented in Python 3.11. As summarized in Figure 4, each plasma event was represented by a background-subtracted 256 × 256 × 3 RGB image acquired from a fixed camera region of interest centered on the laser–aerosol interaction region. The network consisted of three sequential convolutional blocks containing 32, 64, and 128 filters, respectively. Each block comprised a 3 × 3 convolution, rectified linear unit activation, and 2 × 2 max pooling. The successive pooling operations reduced the spatial dimensions of the feature maps while retaining the dominant plasma morphology and channel-dependent intensity distributions. After the third convolutional block, global average pooling converted the final feature maps into a 128-element feature vector. This vector was passed through a fully connected layer containing 32 units and then through a single-output layer that generated one logit. During inference, the logit was converted using a sigmoid function to produce an event-level Cs-likeness score, *s_i_*, ranging from 0 to 1.

The network was trained using binary cross-entropy with logits and the AdamW optimizer. The initial learning rate, weight decay, and batch size were 10^−3^, 10^−4^, and 32, respectively. Training was performed for a maximum of 100 epochs. Early stopping was applied when the validation loss failed to improve by at least 10^−4^ for ten consecutive epochs, and the model weights corresponding to the minimum validation loss were retained. Model development used only the two end-member conditions, 0% Cs and 100% Cs. Ten independently acquired runs from each condition were divided at the run level into six training runs, two validation runs, and two test runs. After plasma-event filtering, the LiOH-only and CsOH-only datasets contained 10,000 plasma-positive RGB images, respectively. The run-level split was used to prevent correlated frames from the same acquisition run from appearing in multiple subsets. To evaluate the robustness of the model to data partitioning and initialization, the complete training procedure was repeated using 20 different run-level splits and random seeds.

All images from a given acquisition run were assigned to the same subset to prevent closely related frames from appearing in both the model-development and evaluation datasets. Intermediate-composition samples were excluded from training, validation, and model selection and were used only to evaluate the response of the trained model to low-level Cs-containing aerosols. This design allowed the intermediate compositions to serve as completely unseen samples and enabled an independent evaluation of whether the end-member-trained classifier could detect low-level Cs without using those concentrations for model optimization or threshold selection. For the independent end-member test dataset, the CNN achieved an accuracy of 98.5%, sensitivity of 98.0%, specificity of 99.0%, balanced accuracy of 98.5%, and AUROC of 0.998, indicating high classification performance for independently acquired plasma images not used during model development.

To further examine the relative contributions of color and spatial information, two additional baseline analyses were performed using the same run-level data partitioning and independent test runs as the original RGB CNN. For the color-only baseline, each plasma image was spatially integrated into three global R, G, and B features and classified using logistic regression, thereby retaining color information while removing spatial structure. For the spatial-only baseline, the RGB images were converted to grayscale and evaluated using the same CNN architecture, retaining the two-dimensional plasma morphology while removing color information. The color-only logistic-regression model achieved an accuracy of 81.2%, whereas the grayscale CNN achieved 70.1%, compared with 98.5% for the original RGB CNN. These results suggest that neither globally integrated color information nor grayscale spatial information alone was sufficient to reproduce the performance of the full RGB CNN, indicating that the combined use of color and spatially resolved image information contributed to the improved classification performance.

For each plasma-positive event, the sigmoid output *s_i_* was interpreted as a Cs-likeness score. Scores approaching zero indicated greater similarity to the Cs-negative reference distribution, whereas scores approaching one indicated greater similarity to the Cs-positive reference distribution. The score was not interpreted as a direct estimate of the Cs fraction in an individual event. Because Li and Cs concentrations varied simultaneously in the present experimental design, this score represents the overall transition from the LiOH reference toward the CsOH reference and should not be interpreted as a uniquely Cs-specific response. Future studies could further investigate the chemical specificity of the present approach by introducing additional aerosol species and independently varying the Li and Cs concentrations to decouple their respective contributions to the RGB and CNN responses.

To reduce the influence of event-to-event variability, consecutive laser shots were divided into 100-shot measurement blocks. The block-level score, S_100_, was calculated as the mean Cs-likeness score of the plasma-positive events within each 100-shot block

(1) $$S_{100}=\frac{1}{N_{plasma}}\sum_{i=1}^{N_{plasma}}s_{i}.$$

The decision threshold, T, was established exclusively from 100 independent 100-shot blocks obtained from the LiOH-only validation dataset. Because these blocks represented the Cs-free background, a block classified as Cs-positive corresponded to a false-positive indication of Cs. The threshold was defined as the 99th percentile of the Cs-free S_100_ distribution, yielding T = 0.13 and an expected false-positive rate of approximately 1% under the present measurement conditions. The threshold was fixed before evaluating the intermediate-composition samples. A measurement block was classified as Cs-positive when S_100_ ≥ T and as Cs-negative when S_100_ < T.

### 3.4. Cs Aerosol Detection Performance and Operational Limit of Detection (LOD)

**Figure 5:**
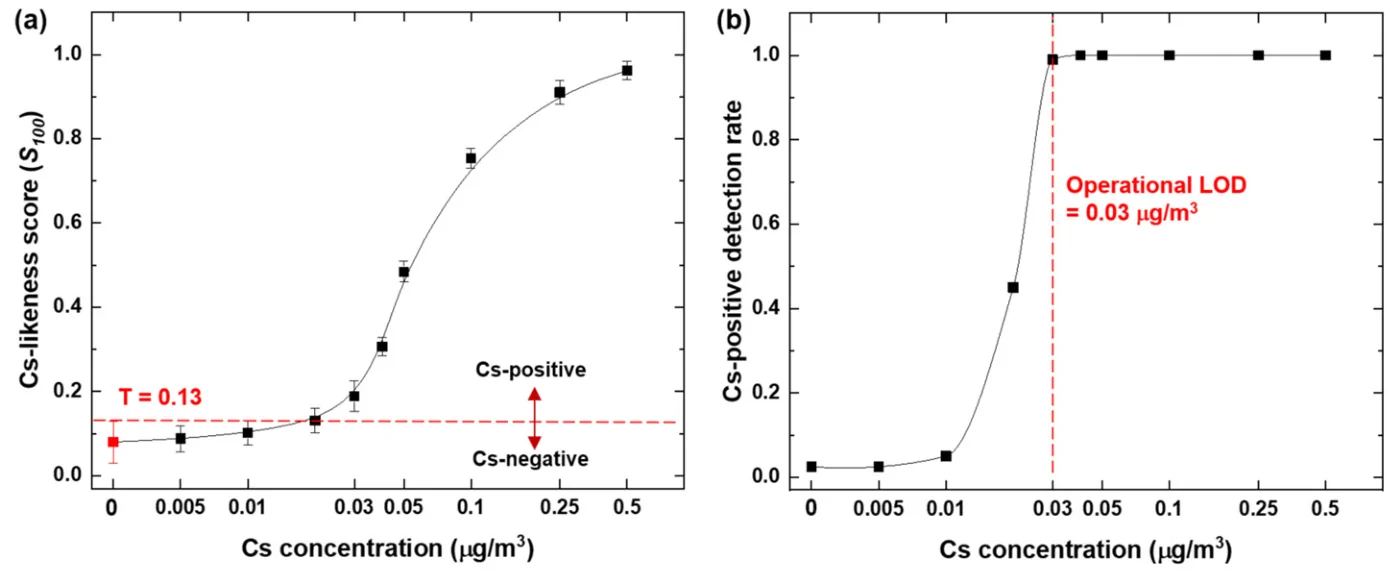
Concentration-dependent CNN response and operational limit of detection for Cs-containing aerosols. (**a**) Aggregated Cs-likeness score, S_100_, obtained from 100-shot measurement blocks as a function of Cs concentration. The horizontal dashed line indicates the decision threshold, T = 0.13. Measurement blocks with S_100_ ≥ T were classified as Cs-positive, whereas those with S_100_ < T were classified as Cs-negative. Symbols represent the mean S100 values from 100 independent measurement blocks, and error bars indicate standard deviation. The smooth curves in both panels were generated using spline interpolation as guides to the eye and were not used for quantitative analysis or determination of the operational LOD. (**b**) Cs-positive detection rate, calculated as the fraction of 100 independent measurement blocks classified as Cs-positive at each Cs concentration. The operational limit of detection (LOD) was defined as the lowest tested concentration at which at least 99 of 100 blocks were classified as Cs-positive. Based on this criterion, the operational LOD was determined to be 0.03 μg/m^3^, as indicated by the vertical dashed line.

The trained CNN and predefined decision threshold were applied to the mixed-composition aerosol samples. Figure 5a shows the resulting concentration-dependent response of the aggregated Cs-likeness score, S100. Each point represents the mean S100 obtained from 100 independent 100-shot measurement blocks, and the error bars represent ±1 standard deviation. The smooth curves were generated using spline interpolation only as guides to the eye and were not used for quantitative fitting or determination of the operational LOD.

The Cs-free (LiOH-only) blocks were concentrated predominantly below the decision threshold of T = 0.13, whereas the S_100_ distributions progressively shifted toward higher values with increasing Cs concentration. This represented the similarity of the color and spatial characteristics of the plasma images to those learned from the CsOH condition. This trend was consistent with the redistribution of the RGB-channel contributions observed in Figure 3. Increasing the Cs content decreased the relative red-channel contribution and increased the blue-channel contribution, shifting the plasma-image characteristics toward those of the CsOH-only end member. The threshold converted the S_100_ response into a binary decision, as Cs-positive when S_100_ ≥ 0.13 and as Cs-negative when S_100_ < 0.13.

Figure 5b presents the Cs-positive detection rate obtained from 100 independent measurement blocks at each concentration. The detection rate increased with increasing Cs concentration.

For the present binary sensing framework, the operational limit of detection (LOD) was defined as the lowest experimentally evaluated concentration that satisfied the predefined 99% detection criterion under the 100-shot measurement protocol. This operational LOD is conceptually different from a conventional analytical LOD derived from a calibration curve. Because 0.03 μg/m^3^ was the lowest tested concentration satisfying the criterion, whereas 0.02 μg/m^3^ did not, the exact detection transition is experimentally bracketed between 0.02 and 0.03 μg/m^3^. Based on this predefined criterion, the operational LOD was determined to be 0.03 μg/m^3^.

The proposed approach combines a low operational LOD with a short measurement interval and a simplified optical configuration. Each sensing decision was obtained from 100 consecutive laser shots, corresponding to 5 s of data acquisition at a repetition rate of 20 Hz. Unlike conventional laser-based elemental analysis, which generally requires wavelength-resolved plasma-emission measurements using a spectrometer, the present method extracted composition-sensitive information directly from broadband RGB plasma images. Eliminating the spectrometer substantially simplifies the detection configuration and facilitates implementation using compact imaging devices. The combined laser-induced plasma RGB imaging and CNN framework therefore provides a rapid, sensitive, and spectrometer-free strategy for detecting trace Cs-containing aerosols.

The present experiments employed a simplified two-component LiOH/CsOH aerosol in N_2_ to demonstrate the feasibility of image-based Cs discrimination. At the selected laser energy of 5.0 mJ, clean N_2_ produced negligible breakdown, indicating that plasma initiation was dominated by the aerosol particles under the present conditions. Nevertheless, N_2_ and other gas species may contribute to the plasma background and influence plasma evolution after breakdown occurs. In actual severe-accident conditions, additional aerosol species and gas components may alter the plasma formation, broadband emission, and RGB intensity distribution. In particular, Na-, K-, Rb-, and Sr-containing species, as well as structural-material aerosols such as Fe- and Ni-containing particles, may produce overlapping emission contributions within the broad RGB channels. Therefore, the present classifier should not yet be interpreted as universally Cs-specific under arbitrary aerosol conditions. Accordingly, further validation and retraining using more representative multicomponent aerosols and accident-relevant gas compositions will be required before practical application.

In addition, the operational LOD reported here is specific to the aerosol-generation conditions used in this study. At a given airborne Cs mass concentration, the detection response may also depend on aerosol number concentration, particle-size distribution, Cs loading per particle, and the degree of internal or external mixing among aerosol species. Heterogeneous particle composition may further modify plasma formation and emission through matrix effects, as previously reported for aerosol LIBS measurements [20]. Future studies could extend the present framework to more representative mixed aerosols and potential interferents, including variations in particle size, composition, and matrix conditions, to evaluate the robustness and general applicability of the method under more complex aerosol environments. A stepwise validation strategy will include individual interferent aerosols, followed by binary Cs/interferent mixtures and progressively more complex multicomponent aerosols, to evaluate cross-sensitivity and determine whether additional retraining or calibration is required.

## 4. Conclusions

This study demonstrated a compact approach for detecting Cs-containing aerosols by integrating laser-induced plasma RGB imaging with CNN-based analysis. The breakdown probability exhibited little dependence on aerosol composition and therefore provided insufficient chemical selectivity on its own. In contrast, the RGB plasma images retained subtle composition-dependent color and spatial information that could be extracted by the CNN. Trained using LiOH-only and CsOH-only plasma images, the CNN converted these features into an event-level Cs-likeness score. Aggregating the event-level scores over 100 consecutive laser shots reduced shot-to-shot variability and enabled binary discrimination between normal Cs-free and abnormal Cs-positive conditions. A decision threshold of T = 0.13, established as the 99th percentile of the Cs-free validation response, constrained the expected false-positive rate to approximately 1%. Under the predefined 99% detection criterion, the operational limit of detection (LOD) was determined to be 0.03 μg/m^3^. These results demonstrate that direct RGB plasma imaging combined with CNN-based classification can provide rapid and highly sensitive detection of low-concentration Cs-containing aerosols while retaining a simplified, spectrometer-free configuration and a straightforward binary sensing output. Consequently, the proposed framework provides a promising basis for compact, near-real-time monitoring of Cs-containing aerosols in nuclear facilities and other environments requiring rapid detection of abnormal aerosol releases.

## Figures and Tables

**Table 1:** CsOH/LiOH aerosol compositions used to evaluate Cs detection. The total airborne alkali-metal concentration was maintained at 0.50 μg/m^3^, while the relative contributions of Li and Cs were varied from the LiOH-only condition to the CsOH-only condition.

Sample	Cs Fraction (%)	Cs (µg m^−3^)	Li (µg m^−3^)
1	0	0.000	0.500
2	1	0.005	0.495
3	2	0.010	0.490
4	4	0.020	0.480
5	6	0.030	0.470
6	8	0.040	0.460
7	10	0.050	0.450
8	20	0.100	0.400
9	50	0.250	0.250
10	100	0.500	0.000

## Data Availability

The data presented in this study are available on request from the corresponding author.
